# AC/off-grid photovoltaic powered open-source ball mill

**DOI:** 10.1016/j.ohx.2023.e00423

**Published:** 2023-04-20

**Authors:** Maryam Mottaghi, Motakabbir Rahman, Apoorv Kulkarni, Joshua M. Pearce

**Affiliations:** a Department of Mechanical and Materials Engineering, Western University, London, Canada; b Department of Electrical and Computer Engineering, Western University, London, Canada; c Department of Electrical and Computer Engineering, Ivey Business School, Western University, London, Canada

**Keywords:** Ball mill, Grinding, Mechanical grinding, Open hardware, Open-source hardware, 3-D printing

## Abstract

Ball milling is used for comminution by rotating a drum to grind materials using balls with specific diameters. Ball milling advantages include the potential for high capacity, predicted fineness in a specific amount of time, reliability, safety, and simplicity, but has disadvantages of high weight, energy consumption and costs, which limit accessibility. To overcome these limitations this study applies the free and open source hardware approach coupled to distributed digital manufacturing to fabricate a ball mill with a simple, customizable design that can be used in a wide range of scientific applications and circumstances including those without access to reliable grid electricity. The highly-customizable design reduces the cost to <US$130 for an AC powered version and <US$315 for a switchable power that enables off-grid operation with a solar module and battery. Using a solar photovoltaic energy source not only improves the power reliability, but also makes it easier to move the ball mill for use in field environments. The open source ball mill is capable of reducing silicon particle sizes from the millimeter scale down to the nanometer scale.

## Graphical abstract

**Figure:**
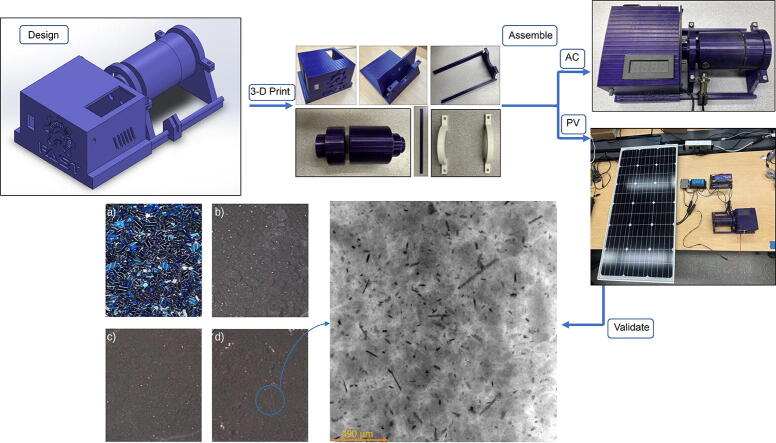

## Nomenclature

Abbreviation: Definition
ABS: Acrylonitrile Butadiene Styrene
AC: Alternating Current
BOM: Bill of materials
CAD: Computer Aided Design
DC: Direct Current
DPDT: Double pole double throw
DIY: Do it yourself
FOSH: Free and Open Source Hardware
IEA: International Energy Agency
OSF: Open Source framework
PLA: Polylactic Acid
PV: Photovoltaic
PVC: Polyvinyl Chloride
PWM: Pulse Width Modulation
RepRap: Replicating Rapid Prototyper
RPM: Revolutions Per Minute
STEP file: Standard for the Exchange of Product model data file
STL file: Stereolithography file

**Specifications table**

**Table:** 

Hardware name	*Open source ball mill*
Subject area	• Engineering and materials science
Hardware type	• Mechanical engineering and materials science
Closest commercial analog	*Commercial Laboratory Ball Mills range from https://www.amazon.ca/Crabby-Laboratory-Driven-Stainless-2KG-SS/dp/B0988BF4BL] CAD$350–[*https://www.coleparmer.ca/i/cole-parmer-jar-mill-without-jars-115–230-vac-50–60-hz/0417250*] CAD$6,775*
Open source license	*Documentation:* GNU General Public License (GPL) 3.0; Hardware: CERN OHL-S v2
Cost of hardware	CAD$170 (AC powered); CAD$420 (AC and off-grid DC with solar power and battery)
Source file repository	*https://osf.io/xa4ws/*
OSHWA certification UID	CA000026

### Hardware in context

Comminution is used to reduce the particle size, change the shape of particles, eliminate agglomeration, provide mechanical alloying, mixing, changing materials properties, and producing powder [1]. There are different grinding techniques such as rod mills, vibrating grinders, medium agitating mills, jet mills, and ball-medium types. The latter one has different methods based on the motion mode of the mill body including tumbling ball mills, vibrating mills, cylindrical ball mills, conical ball mills, and planetary mills. Ball milling is a technology used for grinding, preparing, and modifying materials. The purpose of the ball milling is to rotate a drum with a motor and grind the inside materials using balls with specific diameters. Ball milling has applications in different scientific areas, including chemistry for fabricating microstructures [2], nanostructures [3], [4], [5], and chemical and mechanochemical synthesis [6], [7], [8], [9]. The advantage of ball milling compared to other comminution methods are the potential for high capacity, predicted fineness in a specific amount of time, reliability, safety, simplicity, and servicing [1]. Ball milling, however, has disadvantages, including high weight, high energy consumption because of the waste of energy in heat, friction, and sound [1], as well as high cost (Table 1). Although commercial ball mills can satisfy these applications, their costs are limit accessibility in many labs as they range from approximately US$350 to over US$50,000 for simple laboratory ball mill.

**Table 1:** Commercial ball mill costs in CAD.

Commercial Proprietary Product	Cost (CAD$)	Specifications
Laboratory Ball Mill [10]	349.00	• Capacity: 2 kg • Material: Stainless Steel • Dimensions L × W × H: 46.5 × 26 × 26 cm • Weight: 5 kg
Ball Mill 2 kg Heavy Duty IN 220 Volt Laboratory Ball Grinder [11]	836.00	• Capacity: 2 kg • Material: Stainless Steel • Speed: 80 rpm
One-tier jar high-capacity laboratory jar mill [12]	4,891.65	• Capacity: 27 kg • Dimensions H × W × D: 26.9 × 26.7 × 25.4 cm • Speed: 20 to 300 rpm
Cole-Parmer Jar Mill [13]	6,665.62	• Capacity: 13 kg • Dimensions H × W × D: 15.2 × 34.3 × 25.4 cm • Material: Steel • Speed: 10 to 260 rpm

High costs for scientific equipment like ball mills has been a historic issue as it limits access to scientific tools and drives inequity [14]. Fortunately, there is a proven approach to reducing the cost of scientific equipment is to apply free and open source hardware (FOSH) technological development models [15], [16], [17]. Most recently a review found for a wide range of scientific tools, that open source technologies provide economic savings of 87% compared to equivalent or lesser proprietary tools [18]. These economic savings increased slightly to 89% for those that used open source electronics like Arduino technology [19], and even more to 92% for those that used RepRap-class 3-D printing [20], [21], [22]. Combining both open source electronics and 3-D printing the savings averaged 94% for free and open source tools over commercial equivalents (or lesser tools) [18]. At the same time, building their own hardware [23], [24], using parametric FOSH [25], [26], allows scientists to build high-quality bespoke research equipment [27], [28], [29], [30]. There has been some development of FOSH tools for mixing including a sample rotator mixer and shaker [31], an orbital shaker [32], a nutating mixer [33], stirring [34], and 3-D programmable shaker [35]. Yet despite many low-cost ball mills published in the DIY and maker grey literature [36], [37], [38], [39], [40], [41], [42], [43], [44], [45], there are no open source ball mills published in the scientific literature, which have been validated. The DIY ball mills available also suffer from several shortcomings. The most important concern about many of these home-made ball mills is that they do not use customized drum in their design and they use the readily available containers with their own specified lid. As a result, they cannot meet the requirements of having standard diameter to height ratio to get the highest-quality results and it is impossible or difficult to customize them to achieve this ratio [37], [39], [42], [45], [46].

To close this gap, this study applies the open-source hardware approach and distributed digital manufacturing to fabricate a ball mill to both reduce costs and provide the user with a simple, customizable design that can be used in a wide range of circumstances including those without access to reliable grid electricity.

### Hardware description

The open-source ball mill developed here is fully customizable and designed to be fabricated with distributed manufacturing. The parametric designs of the main components are 3-D printable on a low-cost readily accessible RepRap-class fused filament 3-D printer, and the electronic parts, bearings, magnets, and balls are provided by a wide-range of of-the-shelf vendors. The design both reduces the mass as well as allows users to customize the volume of the ball mill. Electronic designs are provided to power the device from a AC wall outlet as well as a DC source such as a battery or solar photovoltaic mini-module. Testing and validation are provided to compare the quality of the open-source ball mill with the available commercial ones, and a comparison in price is made to show the economic advantage of the open-source device with commercial peers.

• Reducing the expenses for grinding materials in laboratory experiments including grinding, preparing, mixing, and modifying.
• Customizing the design based on the bespoke needs of the project.
• A low weight, which makes the ball mill portable.
• The possibility of using both AC and a DC photovoltaic mini-module as the power sources.

### Design files

#### Design files summary

##### 3D-printing

**Fig. 1:**
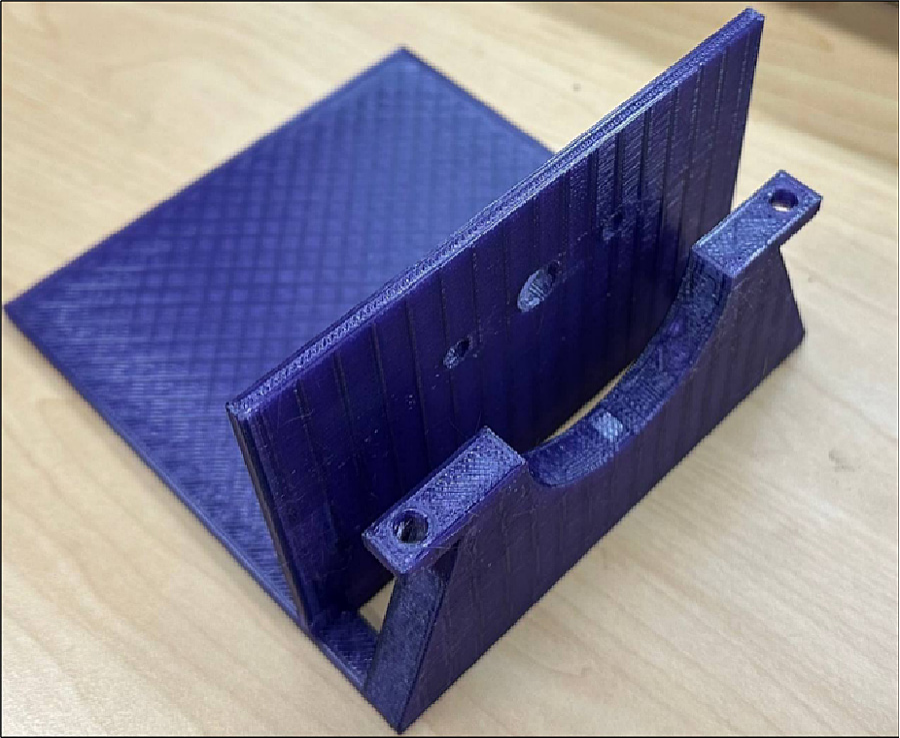
3-D printed base case.

The 3-D-printed hardware is made of several components, including:

File 1 is the 3-D printed base case that accommodates electrical parts (Fig. 1). One bearing should be put on the arc to help the drum rotate easier. Since the system is open source, it can be modified to use any type of bearings and shafts as available. It should be noted, however, that smaller bearing (e.g. low cost 628 skateboard bearings) can only accommodate the shaft and not the drum itself. The reason for using 40 mm inner diameter bearing in this work is to minimize the vibration of the major mass of the system, which is an issue with many of DIY mills developed and tested in the maker community.

**Fig. 2:**
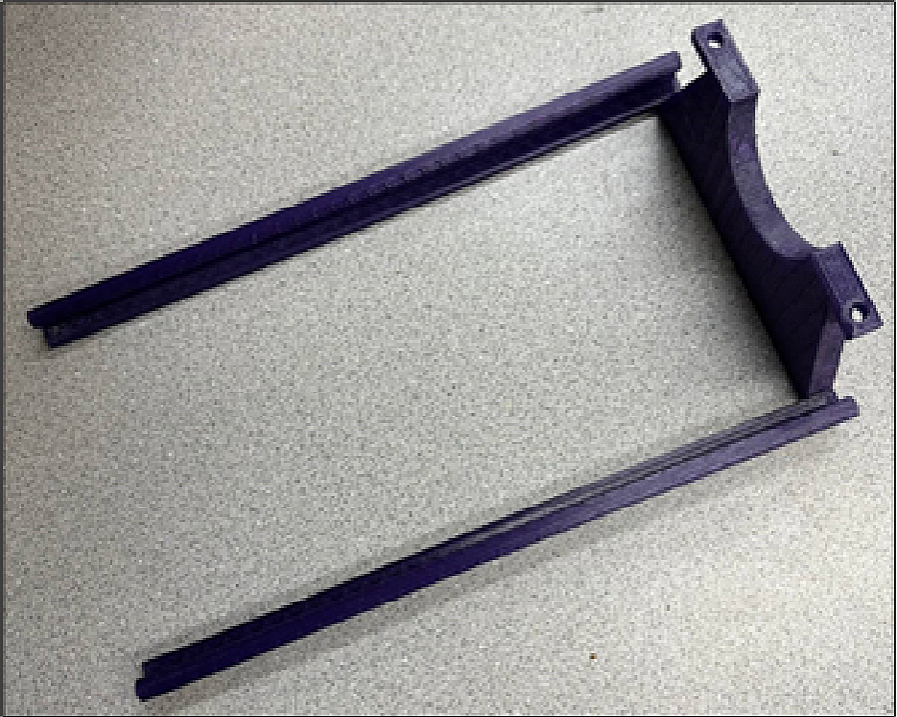
3-D printed front case.

File 2 is the front case (Fig. 2). This part supports the drum mass. One bearing should be attached inside the arc to accommodate the drum and help it rotate easier. Moreover, this part is movable and is adjustable with the drum size.

**Fig. 3:**
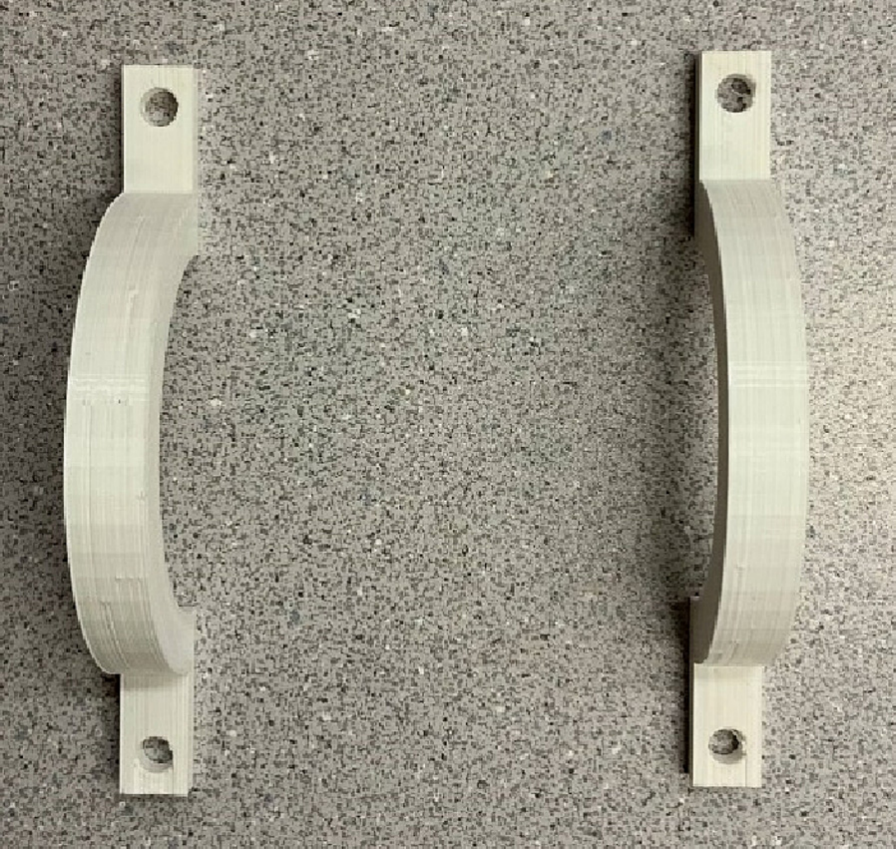
3-D printed locks.

File 3 is the lock, two of which should be attached on top of the front case to lock the drum (Fig. 3).

**Fig. 4:**
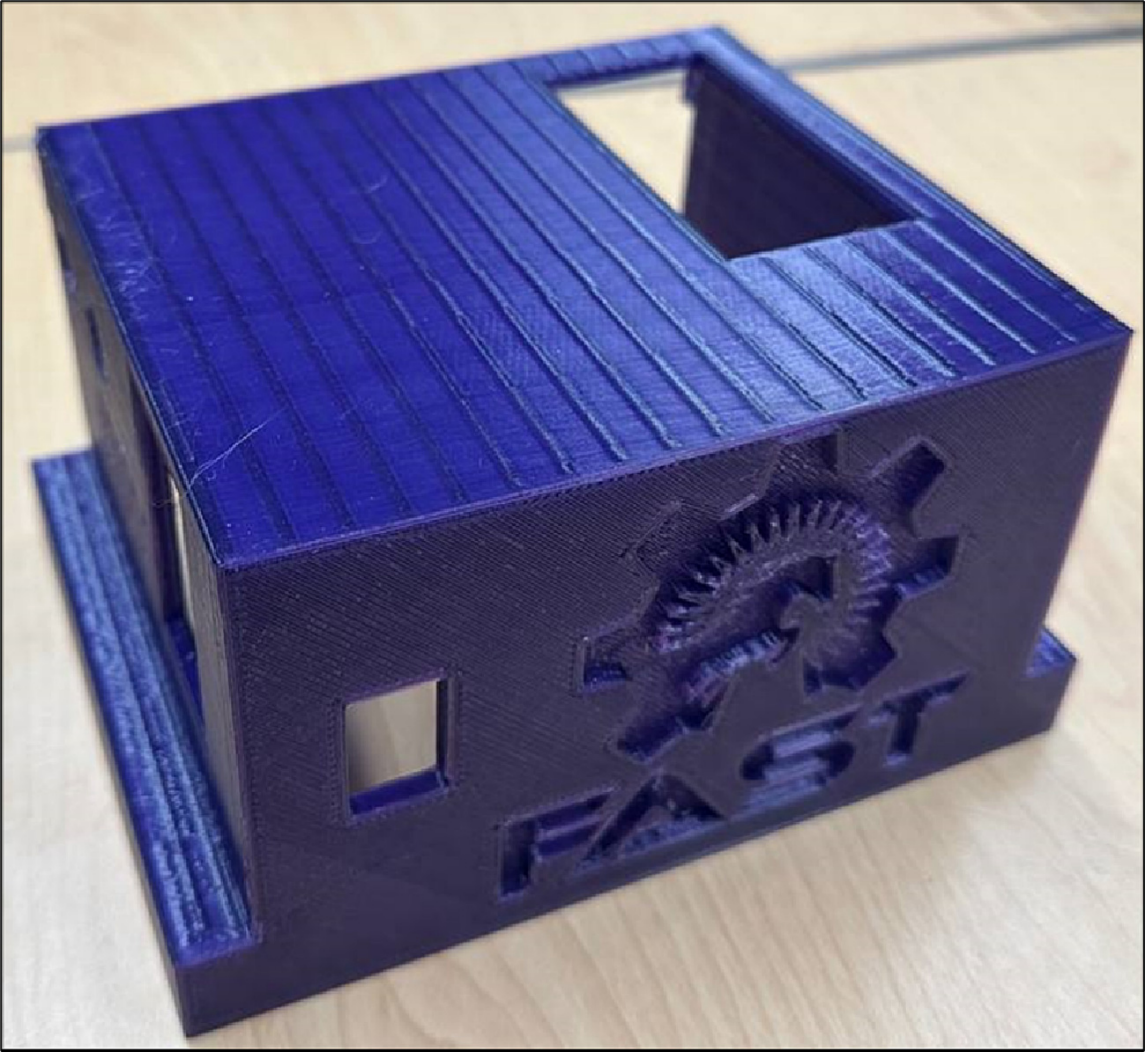
3-D printed cover.

The cover that secures the electrical parts is File 4. Electrical parts including tachometer display, power switch, rotation direction switch, and speed controller knob can be easily integrated into the cover. There are also some ventilation holes on the cover walls that are meant to prevent increasing temperature of electrical parts (Fig. 4).

**Fig. 5:**
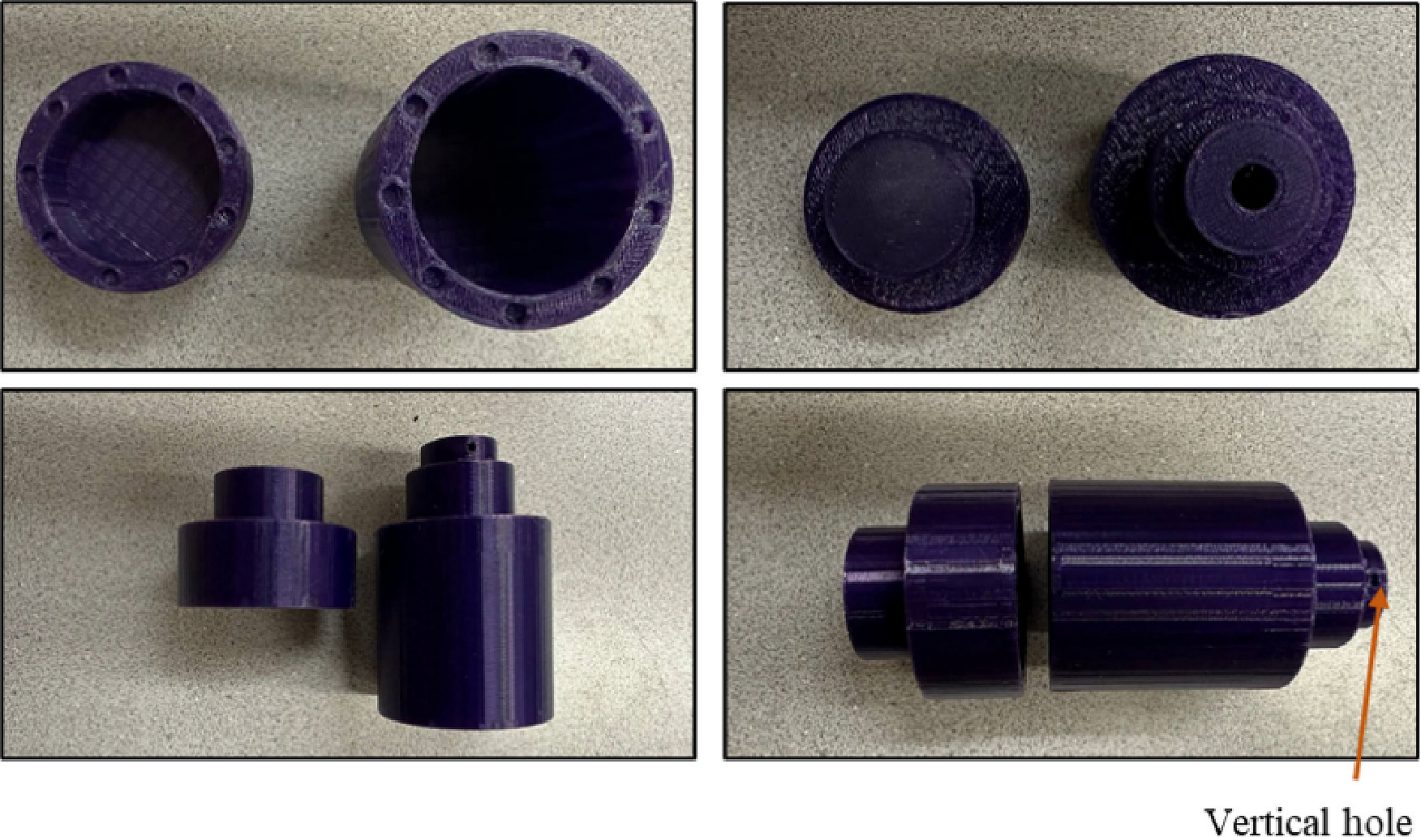
3-D printed drum.

File 5 and File 6 represent the scad format of drum parts that should be filled with balls and materials. In the code, *vf* represents the feed volume and by changing the amount, the design would be changed to the desired size. This part is directly connected to the motor. Considering the potential for pores from 3-D printing inside the drum, one of three methods is needed to smooth the surfaces. First, depending on the 3-D printing material a solvent or vapor polishing can be used for smoothing. There are well established solvent compatibility and chemical compatibility charts [47], [48]. For example, many studies have used acetone on 3-D printed ABS [49], [50], [51], [52]. In this study the most common 3-D printed plastic, PLA, was used and the most common solvent for that is ethyl acetate, which is toxic, carcinogenic and flammable. For these reasons, it is recommended that one of two additional options is used for those working with PLA. The second option is heat treating and is effective at sealing 3-D printed parts for vacuum applications even with a simple heat gun [53]. This approach is effective but takes some practice to get the optimal heat treatment for a specific geometry. A third approach, and the one demonstrated here is to use readily-available PVC pipe located inside the drum to prevent the accumulation of grinded materials inside the pores. This approach has the advantage of being easy to clean if multiple types of materials are to be used in the ball mill. It is worth mentioning that although using a readily available bottles would eliminate the need to purchase magnets, they are not normally applicable since specific diameter to height ratio for the drum is required. In this way, the bottles would normally need to be cut to match the ratio modification, which eliminates the advantage of customizing the drum dimension for different applications. PVC pipes with different ratios, however, are accessible which are convenient to use. Moreover, for grinding the materials where sticking is not a concern, the drum can be used without the PVC. For this purpose, the drum is divided into two parts connected together by magnets. The motor side should be connected to the motor through the hole. Another hole is designed vertically on this side to keep the drum connected to the motor by a set screw. This is shown in Fig. 5.

**Fig. 6:**
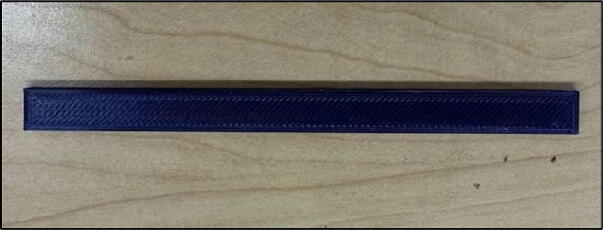
3-D printed cover lock.

File 7 is the cover lock. With this part (Fig. 6), the cover can be attached to the base case.

**Fig. 7:**
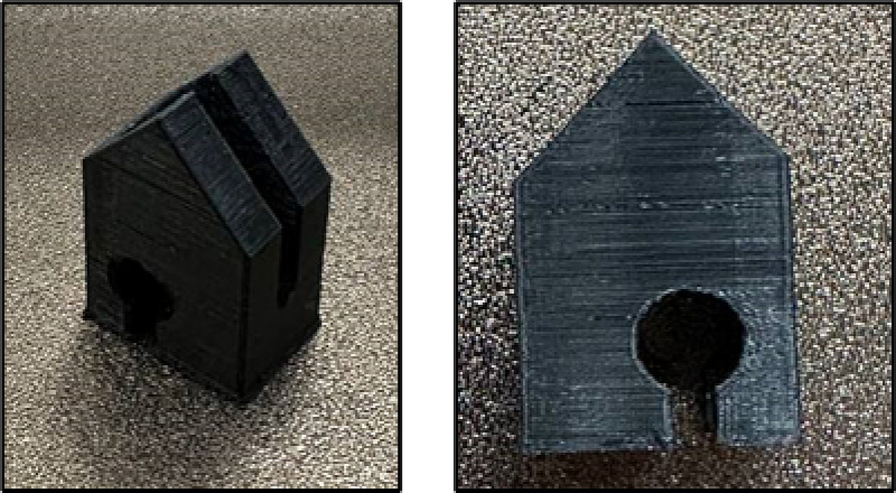
3-D printed speed detector mount.

File 8 is the speed detector mount that can be located on the front case to accommodate the detector in the proper position (Fig. 7).

#### Electrical design with AC power supply

**Fig. 8:**
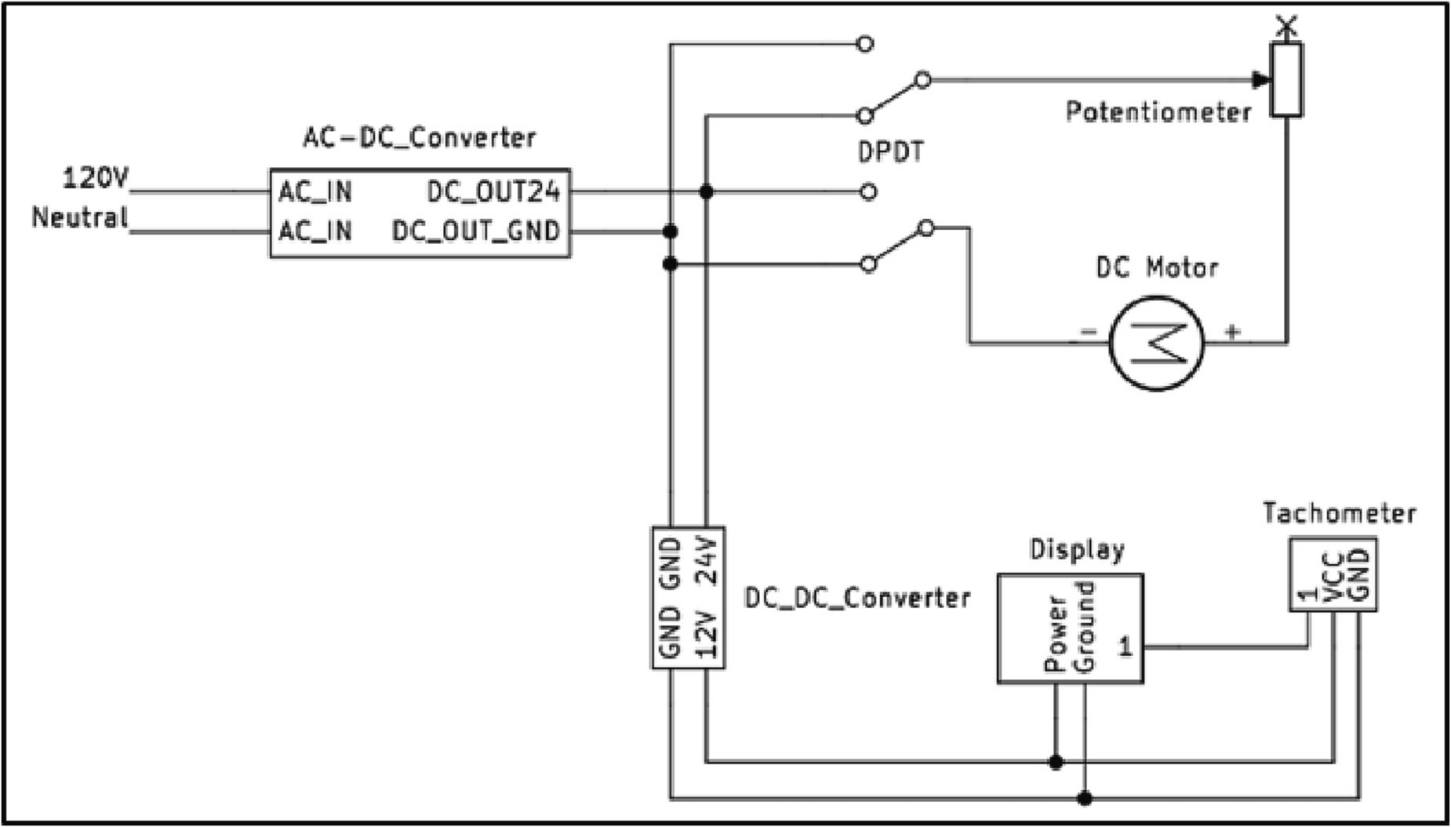
Electrical diagram of AC powered open source ball mill.

The electrical diagram using an AC electric supply as the power source is shown in Fig. 8. The high-speed 30 W permanent magnet DC motor used in this device, can operate at maximum 7000 rpm at 24 V DC. A PWM type DC motor speed controller is connected through a double pole double throw (DPDT) switch, which can regulate the speed as well as reverse the direction of the motor. Both DC motor and speed controller is connected to a 24 V DC supply, which is obtained by converting AC electricity from 120 V AC outlet with an AC-DC converter. Finally, a tachometer with digital display is connected to measure and display the speed of the DC motor. The digital tachometer is capable of measuring speed from 5 to 9999 rpm and operates at 8–15 V DC. The tachometer and the digital display is powered by an additional 24 V to 12 V buck converter, which is connected to the 24 V DC supply.

#### Electrical design with solar photovoltaic power supply

**Fig. 9:**
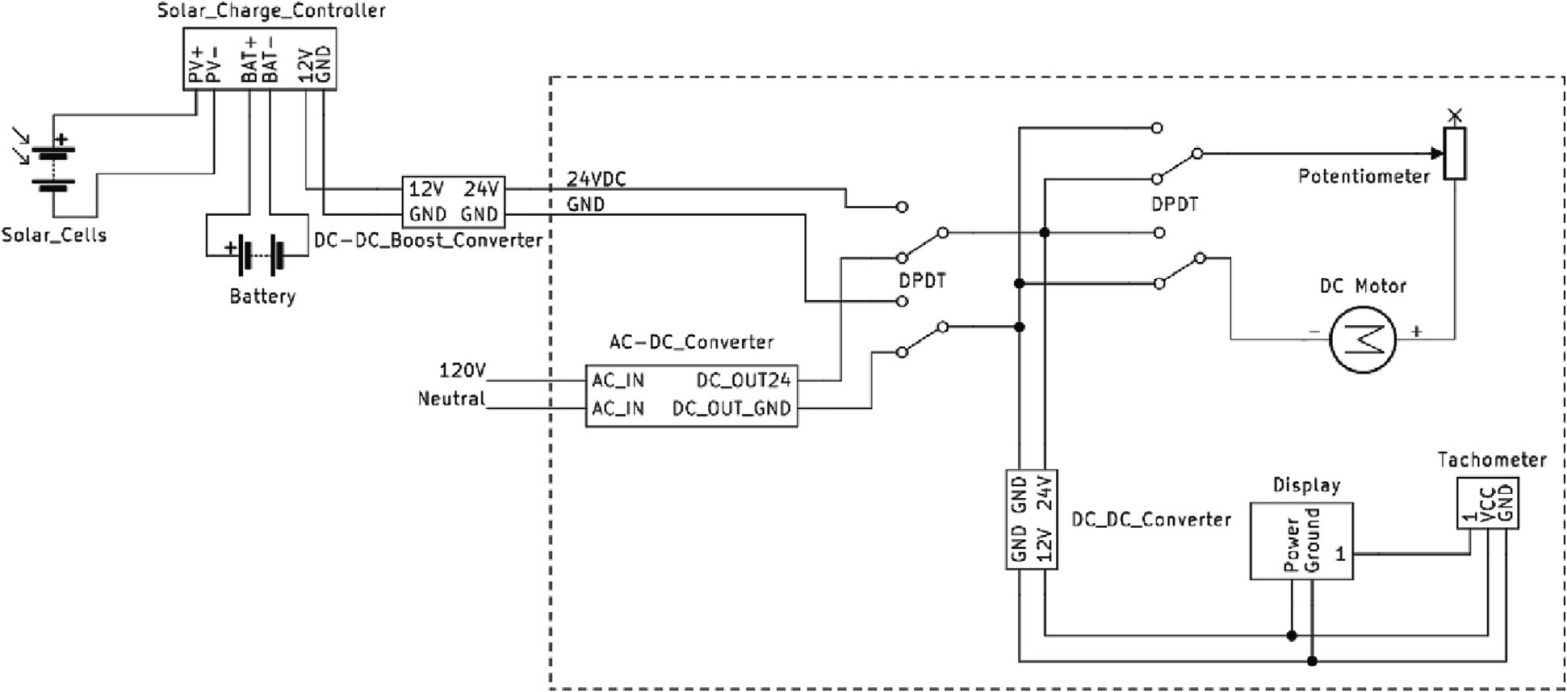
Electrical diagram of upgrade to enable PV powered ball mill.

**Fig. 10:**
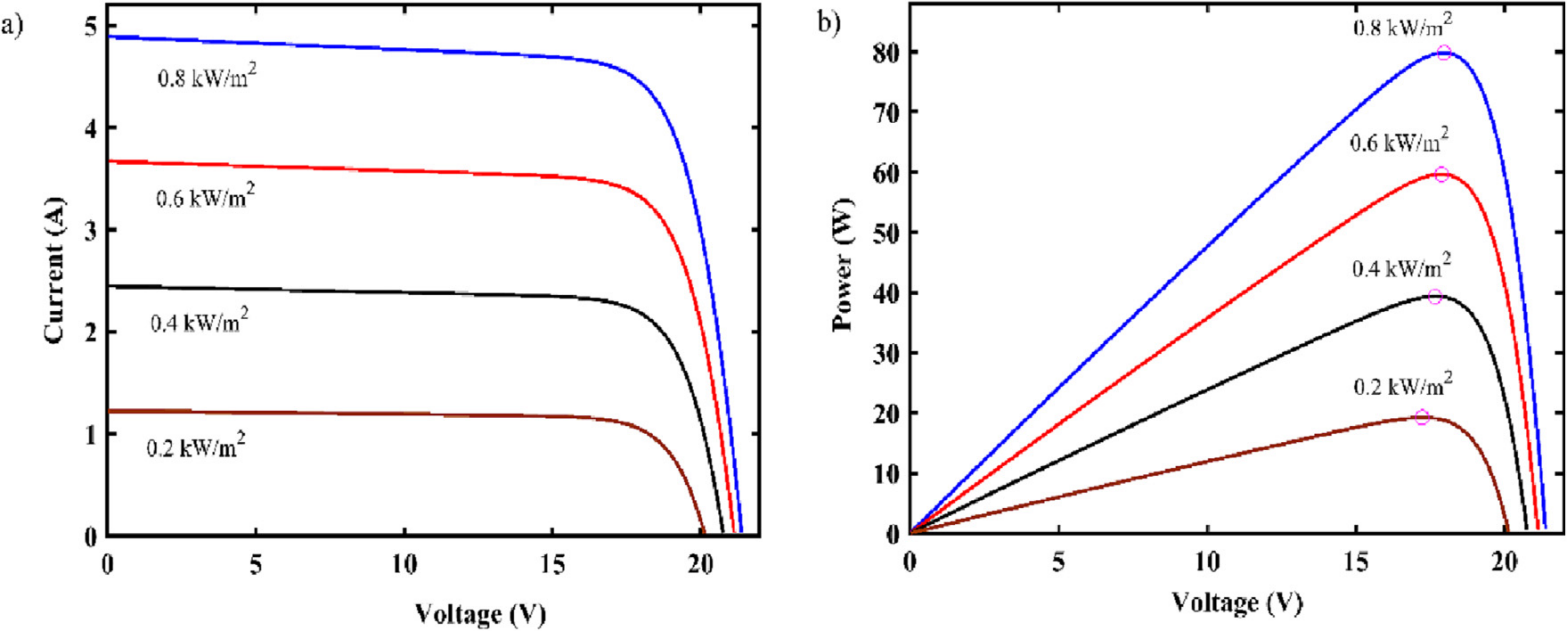
P-V and I-V curves of the solar module under various irradiances (0.8 kW/m^2^, 0.6 kW/m^2^, 0.4 kW/m^2^, 0.2 kW/m^2^).

Moreover, the electrical design shown in Fig. 8 can be modified to operate on solar power by creating an alternative path for 24 V DC supply to the main circuit using a DC connector, which is shown in Fig. 9. In this regard, a DPDT switch is used to isolate the AC interface when a solar photovoltaic (PV) supply is working and vice versa. The 12 V to 24 V converter is used externally to supply the 24 V DC power to the device converting the 12 V supply from the solar PV (see Fig. 10).

As the open-source ball mill is a modest load comprised of a power consumption between 3 and 30 W (due to speed variation), a small PV minimodule of 100 W, with 12 V and 8Ahr battery including a 30A solar charge controller is sufficient to supply DC power to the device with battery backup of almost all day (considering 250 rpm as the usual operation speed).

The solar PV modules has rated power of 100 W with maximum voltage (Vmp) of 18 V, open circuit voltage (Voc) of 21.6 V, short circuit current (Isc) of 6.11A and maximum current (Imp) of 5.55A [54]. It can generate almost 400Whr daily on average. Also from the characteristics curves in Fig. 9, the PV panel can generate minimum of 20 W power if the solar irradiance is 200w/m^−2^ or above. So the standalone PV panel is capable of operating the ball mill and charging the battery under availability of sun.

To provide power backup in absence of solar irradiance, a 12 V 8Ah LiFePO4 battery is used for electric storage. With the depth of discharge of 99%, the battery can provide all day power backup for the ball mill in absence of sun under normal speed of operation. The 30A PWM solar charge controller can supply 12 V DC with maximum discharge current of 10A to the ball mill load effectively. The charge controller will control the charging/discharging of battery as well.

### Bill of materials

#### Bill of materials summary

The BOM of readily available components are shown in Table 3. All the 3-D printed parts are made with an open-source Lulzbot Taz 6 RepRap-class 3-D printer (Fargo Additive Manufacturing Equipment 3D, Fargo, ND, and the material used is a 1.75 mm hard thermoplastic polymer (polylactic acid (PLA)) filament costing CAD$10.83. Other rigid thermoplastic 3-D printing polymers can be substituted in this design. The CAD is available in STL format for SolidWorks, STEP files that can augmented with FreeCAD or OpenSCAD [55], and the slicer for 3-D printing was Cura LulzBot Edition [56]. The electrical components in Table 3 are easily accessible at low prices from a wide variety of internet vendors. The device can be fabricated with the components listed in Table 3 and powered with an AC power source, however, Table 4 represents the electrical parts required for modifying the ball mill so that it can be operated without grid electricity using the solar panel power supply.

**Table 2:** Design file information.

Design file name	File type	Open source license	Location of the file
*Design file 1 (Base case)*	STL, STEP	CERN OHL-S v2	https://osf.io/ahuzq https://osf.io/kqce7
Design file 2 (Front case)	STL, STEP	CERN OHL-S v2	https://osf.io/4hxq3 https://osf.io/yejx8
Design file 3 (Lock)	STL, STEP	CERN OHL-S v2	https://osf.io/qe9ny https://osf.io/4wy9p/
Design file 4 (Cover)	STL, STEP	CERN OHL-S v2	https://osf.io/p7juw https://osf.io/xjr7t
Design file 5 (Drum- motor side)	scad	GNU General Public License (GPL) 3.0, CERN OHL-S v2	https://osf.io/kr5xe
Design file 6 (Drum- far side)	scad	GNU General Public License (GPL) 3.0, CERN OHL-S v2	https://osf.io/tfbwz
Design file 7 (Cover lock)	STL, STEP	CERN OHL-S v2	https://osf.io/svrtc https://osf.io/uh9br
Design file 8 (Speed detector mount)	STL, STEP	CERN OHL-S v2	https://osf.io/gp8q9 https://osf.io/nrxgf
Design file 9 (Open source ball mill operation, loading, unloading)	Video	GNU General Public License (GPL) 3.0	https://osf.io/t7djv https://osf.io/a9kbr https://osf.io/bq6wc

**Table 3:** List of hardware to be purchased for assembly with AC power supply.

Designator	Component	Number	Cost per unit -currency	Total cost – currency	Source of materials	Material type
3D printed case and cylinder	PLA 3-D printing filament	498 gr	CAD$21.99 (per kg)	CAD$10.83	https://www.amazon.com/Polymaker-Filament-1–75 mm-Rigidity-Cardboard/dp/B099JXQN6K/	Polymer
Drive system	Permanent Magnet DC Motor	1	CAD$29.29	CAD$29.29	https://www.amazon.ca/XD-3420-Permanent-Reversible-Electric-Generator/dp/B0BC8KX4HN/	Non-specific
Power supply	24 V AC-DC power supply	1	CAD$16.85	CAD$16.85	https://www.mouser.ca/ProductDetail/MEAN-WELL/IRM-20–24	Non-specific
Drive system	PWM motor speed controller	1	CAD$6.32	CAD$6.32	https://www.amazon.ca/Controller-Control-Reversible-Regulator-Switch/dp/B07QDYPH9G	Non-specific
Drive system	Digital LED Tachometer RPM Speed Meter	1	CAD$24.9	CAD$24.9	https://www.amazon.ca/DIGITEN-Digital-Tachometer-Proximity-Switch/dp/B00VKATA9G/	Non-specific
Drive system	Rocker Switch Power Socket	1	CAD$5.16	CAD$15.49	https://www.amazon.ca/dp/B09BQN1TD9/	Non-specific
Mechanical components	Ball bearing	2	CAD$4.25	CAD$16.99	https://www.amazon.ca/uxcell-6808-2RS-Groove-Bearings-Double/dp/B082PYSPR6/	Metal
Milling media	Balls	2	CAD$17.98	CAD$35.96	https://www.amazon.ca/Breezliy-Piece-Assorted-Bicycle-Bearing/dp/B0982WLFSC	Metal
Mechanical components	Magnets	60	CAD$11.98	CAD$11.98	https://www.amazon.ca/Magnets-Refrigerator-Cylinder-Whiteboard-Miniature/dp/B09CNJ4GN8	Metal
Mechanical components				CAD$168.61

**Table 4:** List of electrical parts to be purchased for assembly with solar mini-module.

Designator	Component	Number	Cost per unit -currency	Total cost – currency	Source of materials	Material type
Solar power generator	100 W Solar Panel	1	CAD$107.99	CAD$107.99	https://www.amazon.ca/ECO-WORTHY-Watts-Volts-Monocrystalline-Solar/dp/B00V4844F4/	Semiconductor
DC energy storage	Lithium (LiFePO4) Battery 12 V 8Ah	1	CAD$69.99	CAD$69.99	https://www.amazon.ca/dp/B092PR8QFQ/	Inorganic
Energy control system	Solar Charge Controller (30A)	1	CAD$23.99	CAD$23.99	https://www.amazon.ca/Controller-Intelligent-Regulator-Paremeter-Adjustable/dp/B08NFSCZ4V/	Semiconductor
Voltage converter	Boost Converter (12 V to 24 V)	1	CAD$32.26	CAD$32.26	https://www.amazon.ca/Converter-10A-Waterproof-Voltage-Regulator/dp/B089M5QBF9/	Semiconductor
Electrical Components for solar module	Toggle Switch	1	CAD$13.99	CAD$13.99	https://www.amazon.ca/WINOMO-Heavy-Toggle-Switch-Waterproof/dp/B075XM68QV/	Metal
Electrical Components for solar module	DC connector	1	CAD$2.74	CAD$2.74	https://www.amazon.ca/Pigtail-Security-Camera-Female-Systems/dp/B092Z6ZG3V/	Metal
				CAD$250.96		

### Build instructions

#### Instructions with AC power supply

The first step for assembling is to 3-D print the components listed in Table 2, which can be found on the Open Source Framework (OSF) [45]. The printing parameters are summarized in Table 5 and can be printed on any RepRap class 3-D printer.

**Table 5:** 3-D printing parameters.

Parameter	Amount
Layer Height	0.18 mm
Initial Layer Height	0.425 mm
Wall Thickness	1 mm
Infill Density	20 %
Infill Line Distance	5 mm
Printing Temperature	210 °C
Build Plate Temperature	60 °C
Print Speed	60 mm/s
Infill Speed	40 mm/s
Wall Speed	30 mm/s
Travel Speed	175 mm/s
Initial layer Speed	15 mm/s
Support density	30 %

**Fig. 11:**
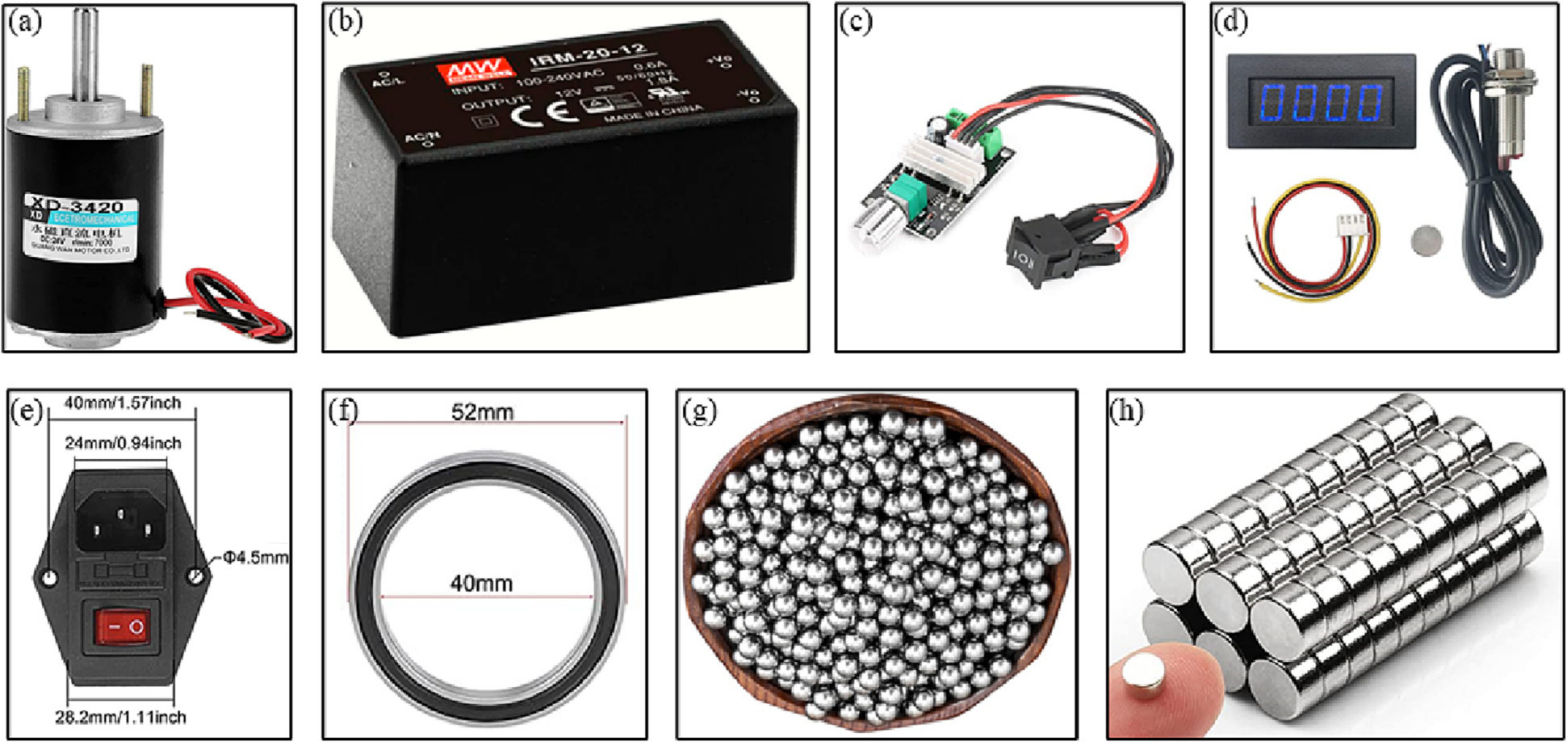
Components to be acquired: a) motor [57], b) power supply [58], c) speed controller [59], d) tachometer [60], e) switch power socket [61], f) ball bearings [62], g) steel balls [63], and h) magnets [64].

Next, the other components from Table 2 must be acquired (Fig. 11).

The assembling steps are as following:

1. Insert electrical parts inside the base case and the cover (Fig. 12). The power socket is placed outside of the cover. Then the AC-DC converter is connected with the 120 V AC supply line. The DC motor and the speed controller are connected in series with 24 V DC. Finally, the tachometer and digital display are connected with the 24 V DC line through a 24 V-12 V buck converter as per the circuit diagram in Fig. 7.

**Fig. 12:**
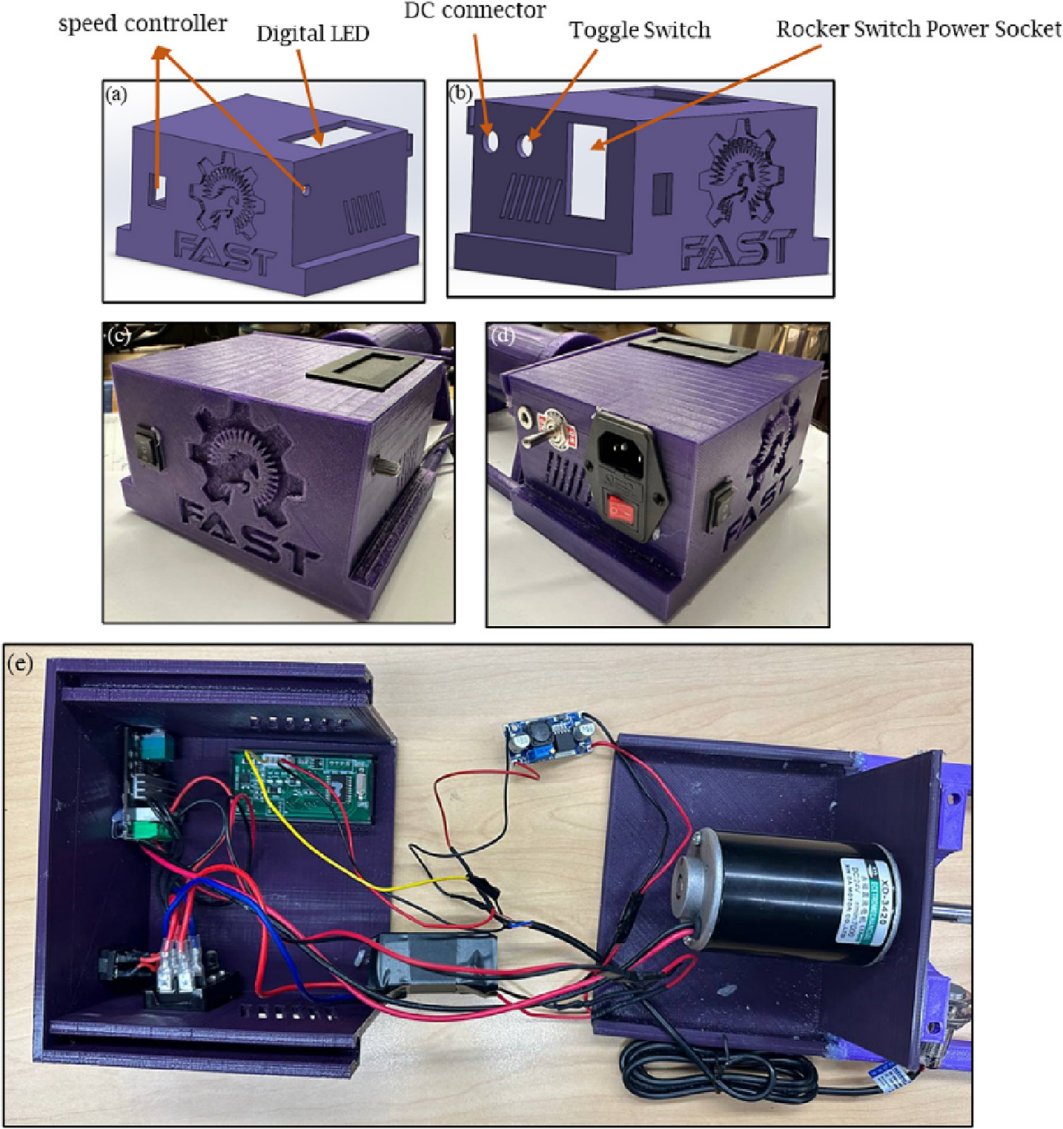
Assembling electrical parts; a and b) position of compounds in the design files, c and d) position of components in the printed parts, and e) full assembly.
2. Insert magnets inside the designed holes in the drum and put the PVC pipe inside the drum (Fig. 13.a and b).

**Fig. 13.O:**
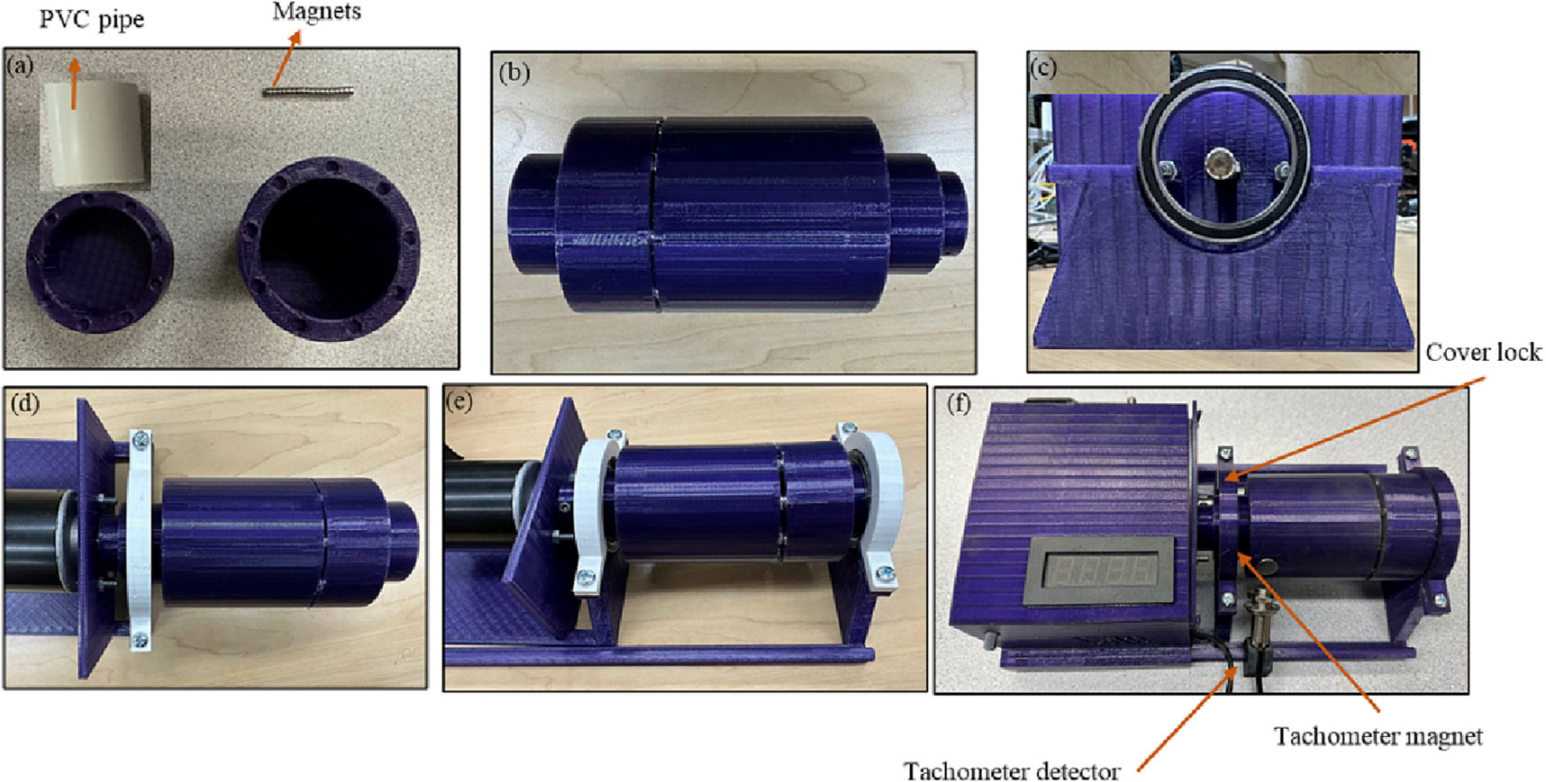
Pen source ball mill assembling steps.
3. Add a bearing inside the arc designed in the base case and secure it with the lock (Fig. 13c).
4. Insert the drum to the motor shaft and secure it by a screw (Fig. 13d).
5. Add the bearing inside the designed area in the front case and secure it with the lock. Insert the far side of the drum into the front case bearing (Fig. 13e).
6. Glue the magnet on the drum body. Then, put the speed detector mount on the front case and insert, and finalize the ball mill (Fig. 13).

The drum rotates through a direct connection to the motor. A pulse width modulation (PWM) circuit board controls the motor speed. Moreover, the tachometer magnet glued on drum body measures and shows the speed on the digital display in RPM. The operation video is provided [65].

#### Instructions with PV power supply

**Fig. 14:**
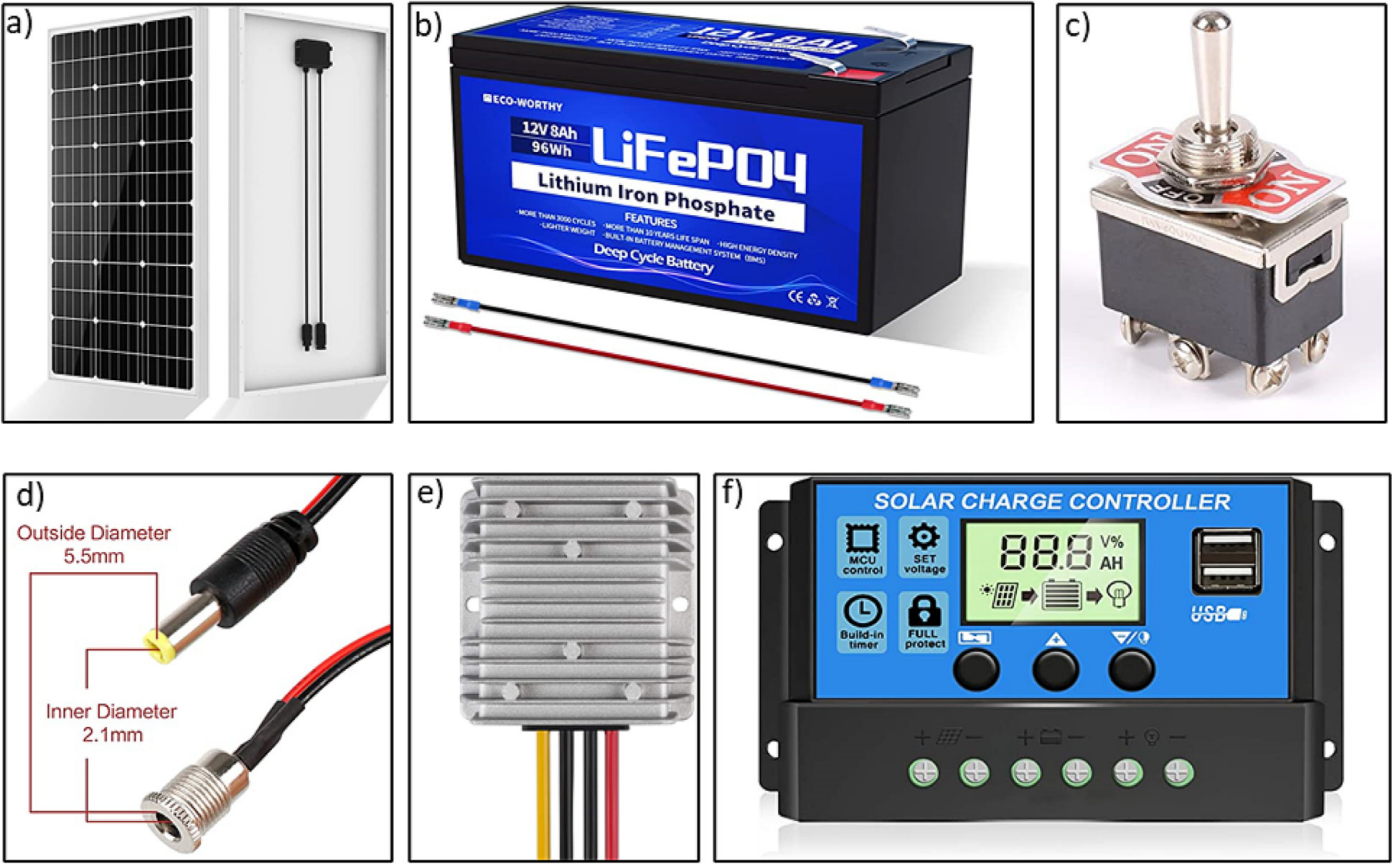
Electrical parts should be acquired for the solar power: a) solar panel [66], b) lithium battery [67], c) boost converter [68], d) toggle switch [69], e) DC connector [70], and f) solar charge controller [71].

For modifying the open source ball mill to be powered by solar energy, the parts from Table 4 should be acquired (Fig. 14).

**Fig. 15:**
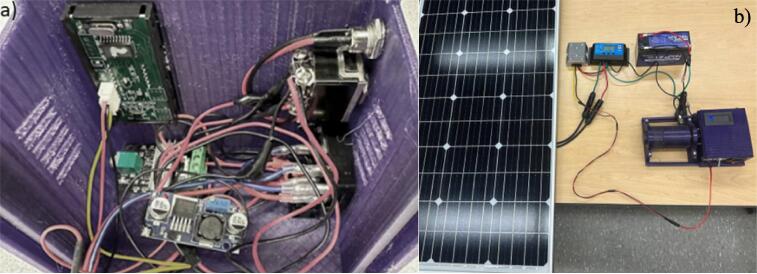
Open source PV powered ball mill assembling, a) modifying the electrical circuit on the cover, b) assembling the PV power source.

The assembling of 3-D-printed parts is similar to the instruction with AC power supply. For the electrical parts, alternative steps are shown in Fig. 15 should be followed as explained below:

1. Modify the electrical circuit inside the base case and the cover according to Fig. 8. The 24 V AC supply is connected with the input pins of the toggle switch. The other two input pins of the toggle switch are connected with the DC connector that supply 24 V DC to the ball mill. The remaining components of the circuit remain unchanged and will be powered by the output pins of the toggle switch. Based on the position of the toggle switch the circuit will be powered by either the AC supply or the solar DC supply.
2. To connect with the PV power, first connect the 12 V, 8Ah battery with the charge controller.
3. Then, connect the 100 W solar panel with the charge controller.
4. Finally, the 12 V DC power converted into 24 V DC by a 12 V-24Vconverter and is supplied to the ball mill via DC connector.
5. To switch the operation between the AC supply and solar power, change the position of the toggle switch shown in Fig. 12.

### Operation instructions

After assembling, the open-source ball mill, it is ready for operation. The first step for operation is to insert materials and balls inside the drum [1]. The ball mill can be turned on by switching the start button to the on position. The speed of the ball mill is controlled by rotating PWM circuit board knob. The speed is shown in the LED display for the tachometer after a few second delay. See video in the OSF directory [65].

For each application the drum dimensions can be customized. The ball mill is designed based on the standard diameter and length to get optimum efficiency. The relationship between the drum volume and the feed volume (V_f_) is as shown in Eq. (1) [72].

(1) $$V_{f}=V_{d}\times 0.25\left[mL\right]$$

Where V_d_ is the drum volume (mL). The optimum ratio between the drum length (L) and the drum diameter (D), L:D, is in the range of 1.56–1.64 [1]. As a result, the drum diameter and length could be calculated by making the drum volume follow Eq. (2).

(2) $$V_{d}=\pi {\left(D/2\right)}^{2}\times L\left[mL\right]$$

Here, the demonstrated system has a drum diameter is 49 mm, and the length is 76.5 mm to accommodate about 36 cubic milliliter of feed volume. The balls that act as grinding media can be from different materials including steel, stainless steel, ceramic, or rubber [4]. It is worth mentioning that grinding materials with steel balls can create Fe contamination [73]. It has been shown that the maximum ball load should not be more than 30–35% of the total volume [1]. Balls with bigger diameter could help to break material and the smaller balls are used for grinding materials to finer particles [74]. In this study, 330 g steel balls including 200 pieces of 6.35 mm (1/4 in.), 200 pieces 5.55 mm (7/32 in.), 100 pieces of 4.76 mm (3/16 in.), and 100 pieces of 3.96 mm (5/32 in.) are used to achieve both goals.

Moreover, Equation (3) represents the relationship between the drum diameter and the critical speed of the drum.

(3) $$n=42.3/\sqrt{D}\left(RPM\right)$$

Where n is the critical speed (RPM) [1]. The critical speed creates a centrifugal force for the balls to rotate inside the drum, travel to the top side of the drum, and fall on the material to grind them through shear and compression forces [75]. It should be considered that the optimum speed of the drum is between 65 and 80% of the critical speed. With speeds less than the optimum, the centrifugal speed is not enough and the balls cannot travel as far up the drum wall to fall down and crush the materials based on their weight. With a speed more than the optimum amount, the centrifugal force is so large that it does not allow the balls to fall and they keep rotating with the drum [1]. As the result, the optimum speed for the 49 mm diameter drum is 191 rpm to grind about 36 cubic milliliters of feed volume.

With the equations in mind, drum diameter, drum length, ball load, ball size, and rotation speed are important factors to consider while designing for the desired use case. The open source ball mill is designed to be customized easily by changing the parameters in the parametric OpenSCAD files.

**Safety**

Safety considerations of the open source ball mill include electrical risks and risks associated with working with powders.

• All the electrical connections must be properly insulated.
• The ball mill should function at a safe environment apart from people or food (e.g. a chemical fume hood). This can be critical depending on the type of material used.
• Use of mask, gloves and safety glasses is required while handling the powders to avoid the fine particles from being inhaled or getting in the user’s eyes.
• Powders should be isolated from other sensitive equipment.
• The users should follow safety guidelines for powders of their specific type and size (e.g. fine powders are often combustible).
• The open source ball mill is designed and validated to be used for dry materials.
• Materials that react with the polymer should not be used.
• The ball mill is validated by Si waste from solar panels, which indicates similar brittle materials like SiC or glass are compatible.

### Validation and characterization

Many research projects utilize ball mills. To demonstrate the effectiveness of the open source ball mill it is validated here by grinding silicon particles from waste solar photovoltaic cells. Other research groups have used ball milling before for this application. For example, Nilssen et al. investigated the properties of silicon after ball milling with a planetary ball mill at different times, ball sizes, and speeds [76]. These parameters affect the phase form, crystalline or amorphous, and the sizes of the silicon particles. Zhu et al. determined the influence of ball milling factors, including milling time and speed, in nano silicon production with zirconia balls. They showed that increasing the speed and milling time increases the rate of breaking particles and results in more evenly particles distribution [77]. In a study by Li et al., the influence of ball size on the particle properties is investigated. They controlled the mass ratio between balls and powder in a 10:1 amount. The results showed that different range of ball sizes from 5 mm to 15 mm diameters could grind the SiC particles from 39.7 and 111 µm to 7.51 and 19 µm, respectively after 5 h [73].

**Fig. 16:**
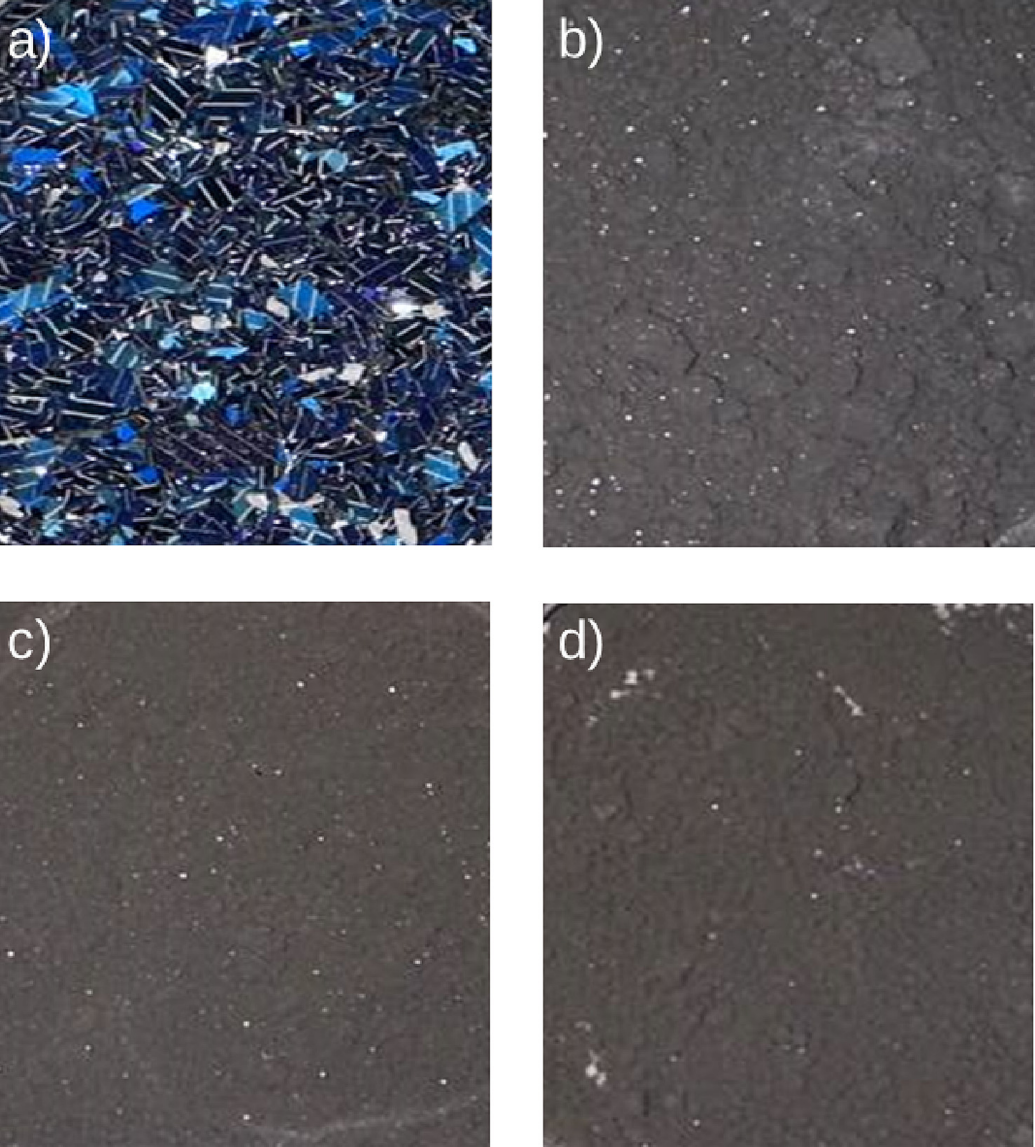
Smart phone pictures of silicon particles: a) before ball milling, b) after 6 h ball milling, c) after 12 h ball milling, and d) after 18 h ball milling.

**Fig. 17:**
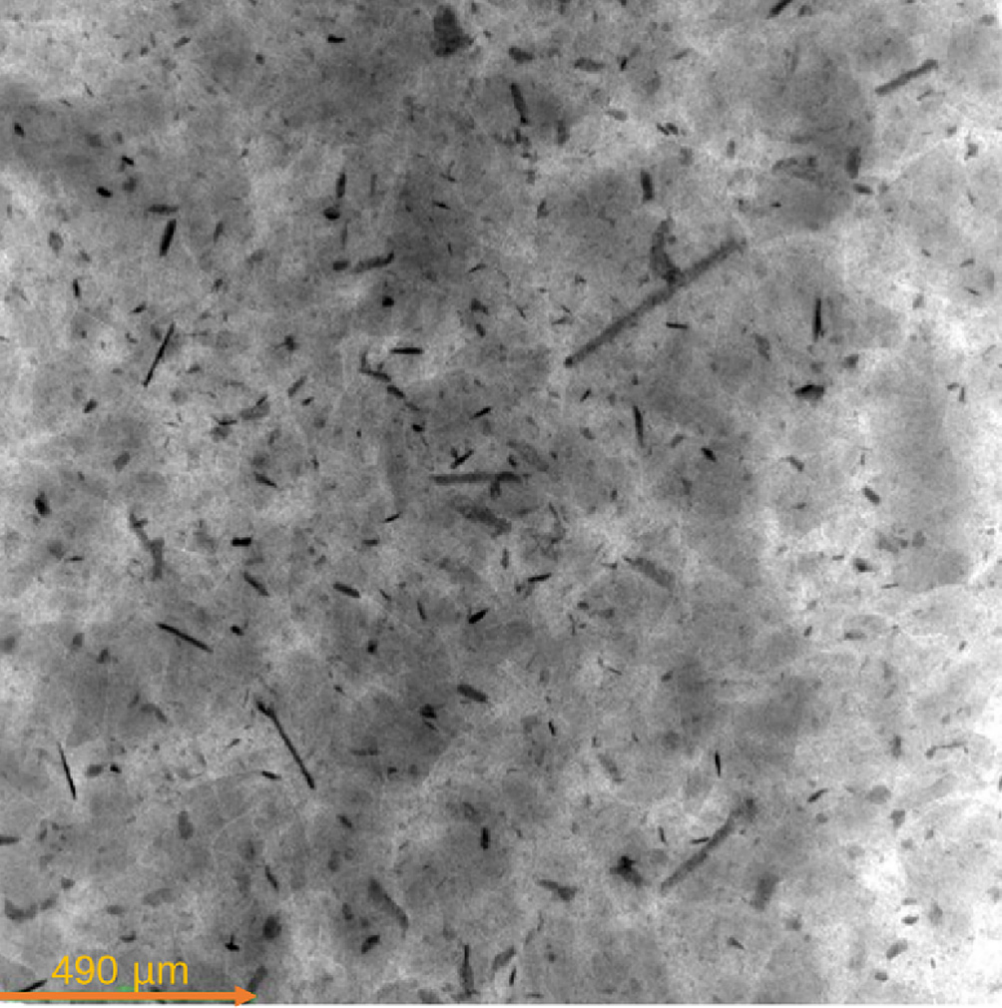
Micro-CT image of silicon particles after 18 h ball milling.

For the open-source ball mill, the ability to grind silicon particles from waste solar photovoltaic cells was tested in detail. The consumed energy was monitored with a digital multimeter (±0.01 kWh) and the final particle sizes for silicon powder are measured using micro-CT. For this purpose, multi-millimeter size silicon wafer shards are added to the drum. It takes about 1 min to load the ball mill. The ball milling was done in four steps with 190 rpm speed. It takes about 40 s for the tachometer to read the speed correctly. For each step, the particles were milled for 6 h, the particle sizes were determined with the use of the micro-CT and the open source imaging processing software Fiji (with ImageJ2). The open source ball mill was run in three sets of six hours and the particle size was measured after each run (Fig. 16 and Fig. 17). The unloading video is provided in OSF directory [78].

**Fig. 18:**
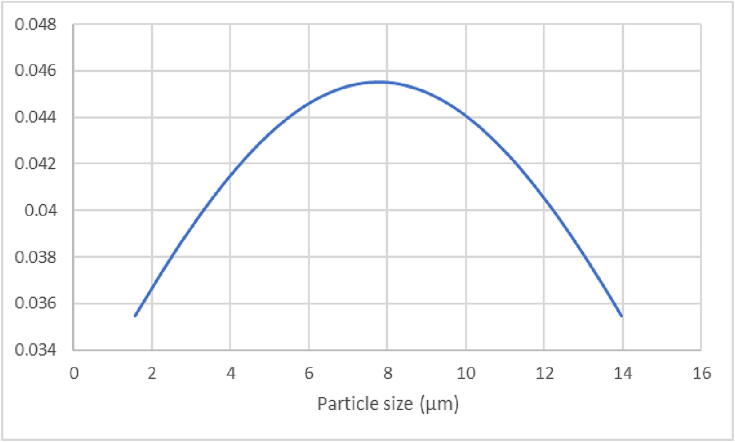
Particle size distribution after 18 h ball milling.

The results show that the silicon particles size reduced from 0.8 mm down to 7 µm 18 h (Fig. 18). As the results clearly show the ball mill is able to grind materials down to micrometer size, which is desirable for many scientific applications.

The capabilities of the open source ball mill include:

• Reducing the cost of ball milling hardware down to CAD$170 for an AC powered device. In addition, the device can be made power switchable to enable off-grid operation with a solar module and battery for CAD$420.
• Using the solar photovoltaic as the energy source not only improves the power reliability, but also makes it easier to move the ball mill for use in field environments.
• Low weight resulting from the structure materials which are 3-D printed plastic also make the ball mill readily transportable.
• The open source ball mill showed that it was capable of reducing silicon particle sizes from mm down to the micrometer scale.
• Finally, it is highly customizable, which helps the user to change the drum size for specific applications and it can be printed out of various materials including a wide range of plastics, composites, ceramics or metals.

One of the limitations of the open source ball mill is that plastic construction might reduce the stability of the ball mill at higher loads (i.e. it is so low weight it might need clamping fixture to secure it to a desktop).

The open source ball mill was shown to be effective, but there are several areas of potential improvement. First, the mass of the plastic parts could be further reduced by iterative design governed by simulations to calculate the stress–strain in different parts and remove the material that are under less stress, to reduce the materials used in the design and make it even more economical. This design could also be built upon by developing an open source planetary ball mill to increase the efficiency. The open source ball mill in the AC version is less costly than the conventional proprietary ball mills, however, when adding the ability for switchable power with a solar photovoltaic mini-module the costs again have become prohibitive for some users. This is primarily because all of the electronic components are off-the-shelf. Future work could focus on making open source equivalents of the solar charge controller and the boost converter. An integrated open source circuit could be developed that replaces all of the electronics for both systems and be available for synthesis using open source circuit mills [79], [80], [81], [82] or electronics 3-D printing [83]. Lastly, it should be pointed out that although the levelized cost of electricity from full-scale solar photovoltaic modules is now often the least costly of electrical sources and the IEA believes the lowest cost in history [84], photovoltaic mini-modules can be significantly more expensive. For example, the spot price on large scale purchases of full-size photovoltaic modules made of high-quality monocrystalline silicon passivated emitter and rear (PERC) cells is $0.235/W on Dec. 10, 2022 [85], while the mini-module used here costs more than $1.00/W. This indicates there is a potential to fabricate open source solar photovoltaic modules that could beat the current costs of even mass-produced mini-modules and there has been some development of open source photovoltaic modules [86], that could be leveraged here to make lower-cost field deployable DIY solar powered instruments.

Finally, future work is needed in the area of material compatibility. Earlier the potential for material contamination was discussed concerning the balls, which is a well-known issue with ball mills of all types and is corrected simply by the material selection of the ball. In this case, for some applications there may also be an issue with the walls of the cylinder (e.g. if polymer contamination is an issue for a particular project). In general, this can be fixed by post-processing the powder with solvents to remove contamination the 3-D printing polymers, which can be easily removed through using appropriate solvent [87].

### Conclusions

To summarize, the highly-customizable, low-weight open-source ball mill is fabricated using low-cost readily accessible RepRap-class fused filament 3-D printers. It can be powered by both an AC power supply as well as a solar PV module and battery with switchable power. The latter enables off-grid operation when proper and reliable grid electricity is not accessible and makes it easier to use the ball mill in the field environment. The final product cost is less than US$130 when using the AC powered version and less than US$315 when is powered by the solar photovoltaic system. The open-source ball mill is applicable for use in a wide range of scientific applications. As and example the open-source ball mill is also made with standard dimensions to work effectively and has been demonstrated to grind silicon particles from waste PV modules from 0.8 mm to 7 µm in 18 h.

## Declaration of Competing Interest

The authors declare that they have no known competing financial interests or personal relationships that could have appeared to influence the work reported in this paper.
